# The regulatory effects of lactic acid on neuropsychiatric disorders

**DOI:** 10.1007/s44192-022-00011-4

**Published:** 2022-03-30

**Authors:** Xueyi Chen, Yangdong Zhang, Haiyang Wang, Lanxiang Liu, Wenwen Li, Peng Xie

**Affiliations:** 1 Department of Pathology, Faculty of Basic Medicine, Chongqing Medical University, Chongqing, 400016 China; 2 NHC Key Laboratory of Diagnosis and Treatment on Brain Functional Diseases, The First Affiliated Hospital of Chongqing Medical University, 1 Youyi Road, Yuzhong District, Chongqing, 400016 China; 3 Department of Neurology, The First Affiliated Hospital of Chongqing Medical University, Chongqing, 400016 China; 4 College of Stomatology and Affiliated Stomatological Hospital of Chongqing Medical University, Chongqing, 401147 China; 5 Department of Neurology, Yongchuan Hospital of Chongqing Medical University, Chongqing, 402160 China

**Keywords:** Lactic acid, Neuropsychiatric diseases, Energy metabolism, Microbiota, Gut, Brain axis

## Abstract

Lactic acid is produced mainly in astrocytes in the brain and serves as a substance that supplies energy to neurons. In recent years, numerous studies identified the potential effects of lactic acid on the central nervous system and demonstrated its role in regulating brain function as an energy metabolism substrate or cellular signaling molecule. Both deficiency and accumulation of lactic acid cause neurological dysfunction, which further lead to the development of neuropsychiatric disorders, such as Major depressive disorder, Schizophrenia, Alzheimer’s disease, and Multiple sclerosis. Although an association between lactic acid and neuropsychiatric disorders was reported in previous research, the underlying pathogenic mechanisms remain unclear. Therefore, an in-depth understanding of the molecular mechanisms by which lactic acid regulates brain function is of significance for the early diagnosis and prevention of neuropsychiatric disorders. In this review, we summarize evidence that is focused on the potential mechanisms of lactic acid as a signaling molecule involved in the pathogenesis of neuropsychiatric disorders and propose a new mechanism by which lactic acid regulates brain function and disease through the microbiota–gut–brain axis to offer new insight into the prevention and treatment of neuropsychiatric diseases.

## Introduction

Lactic acid is a common metabolite in the human body and is considered a waste product that causes fatigue during exercise. In the early nineteenth century, lactic acid was detected for the first time in the muscle tissue of animals after exercise [1]. The traditional theory suggests that lactic acid is a product of energy metabolism and that it participates in redox reactions. However, in the 1970s, a new understanding of lactic acid was developed. George et al. [2] proposed the concept of “the astrocyte-neuron lactate shuttle,” which described lactic acid as not only an important carbon source for aerobic energy metabolism and gluconeogenesis but also a signal molecule that is transmitted to neighboring cells to influence signal communication between cells. Changes in the concentration of lactic acid alter the pH of the body, which affects normal biological reactions in cells and tissues. In recent years, numerous studies showed that lactic acid participates as a signal molecule in the regulation of brain function [3]. In the central nervous system (CNS), lactic acid affects brain function by the corresponding receptors [4]. Thus, abnormal lactic acid metabolism may participate in the development of various neuropsychiatric diseases, such as Major depressive disorder (MDD), Schizophrenia (SCZ), Alzheimer’s disease (AD), Multiple sclerosis (MS), and Myasthenia gravis (MG). Previous literature has confirmed that lactic acid is involved in the development of various common neuropsychiatric disorders. However, the specific mechanism of action remains unclear, and further research is needed. In this review, we summarize the evidence that is focused on the potential mechanisms of lactic acid as a signal molecule and its involvement in the pathogenesis of neuropsychiatric diseases and propose a new mechanism by which lactic acid regulates brain function and disease via the microbiota–gut–brain axis to offer new insight into the prevention and treatment of neuropsychiatric diseases.

## Classification of lactic acid

There are two isomers of lactic acid in the human body: d-lactic acid and l-lactic acid. d-lactic acid exists in micromolar concentrations and accounts for approximately 1% of the concentration of l-lactic acid [5]. l-Lactic acid is primarily derived from the catabolism of carbohydrates and amino acids during the glycolysis process, whereas d-lactic acid is derived from carbohydrate and lipid metabolism [6] as well as intestinal bacteria production [5]. l-Lactic acid and d-lactic acid regulate neural network activity by binding to the hydroxycarboxylic acid receptor 1. l-Lactic acid is the main substrate involved in neural oxidative metabolism. It promotes protein synthesis during learning and memory formation, increases synaptic remodeling and axonal excitability [7], and enhances the formation of memory [8]. In contrast, d-lactic acid can lead to insufficient neuronal energy metabolism and memory impairment due to the competitive blocking of l-lactic acid uptake by neurons [3]. The two types of lactic acid differ in structure, influence on the human body, and mechanism of action; however, most studies do not differentiate between the two types.

## Lactic acid metabolism pathways

In physiological conditions, lactic acid is produced during glycolysis in tissues throughout the body, such as in the muscles, skin, brain, intestines, red blood cells, fat, and other tissues and organs [9], 10. During glycolysis, pyruvate is produced by the oxidation of glucose and reduced to lactic acid that is catalyzed by lactate dehydrogenase (LDH) under hypoxia [4]. A hypoxic state, hyper-activation of glycolysis regulated by the β-adrenergic receptor, or a decrease in lactic acid conversion (e.g., abnormal mitochondrial function and reduced lactic acid clearance) can disrupt the homeostasis of lactic acid, which leads to an increase in the levels of lactic acid in tissues. This subsequently affects the physiological function of the body [11] and in severe cases, acidosis can occur.

### The metabolism and function of lactic acid in the brain

Lactic acid in the brain is produced primarily by astrocytes, and the main production pathways include the glutamate-activated glycolysis pathways and the glycogenolytic pathway, which is activated by norepinephrine, vasoactive peptides, adenosine, and the potassium ion [11]. Lactic acid produced in astrocytes is transported to the interstitial fluid via the monocarboxylic acid transporter (MCT)1/4 on the astrocyte membrane and is subsequently transported into the neuron through the MCT2 on the neuronal membrane [12]. Lactic acid in neurons is then reduced to pyruvate and nicotinamide adenine dinucleotide I (NADH I) via catalysis by LDH1 [4]. NADH enhances the calcium current by binding to *N*-methyl-d-aspartate (NMDA) receptors, which subsequently activate intracellular signal cascades and upregulate the expression of genes related to neuroplasticity (e.g., activity-regulated cytoskeleton-associated protein, early growth response protein 1, and brain-derived neurotrophic factor [BDNF]). Pyruvate enters the neuronal mitochondria through the MCT and catalyzed to generate acetyl coenzyme A by pyruvate dehydrogenase, which enters the tricarboxylic acid cycle [11] to provide energy for neurons. Lactic acid in the brain is mostly cleared after being transported into the cerebrospinal fluid via perivascular transport; however, during this process, a small amount is re-uptaken into the brain through the blood–brain barrier (BBB) [13]. Under physiological conditions, lactic acid in the brain acts as an energy metabolism substrate to participate in neuronal energy supply, or as a signal molecule to participate in the regulation of brain function, promote protein synthesis during learning and memory, increase synaptic remodeling and axon excitability, and enhance the formation of memories. In pathological conditions, the impaired BBB increases the re-uptake of lactic acid by 30% [13], which leads to reduced lactic acid clearance [14]. The decreased expression of G protein-coupled receptor 81 (GPR81) and MCT1 in brain microvessel endothelial cells causes significant impairment in the integrity and increases the permeability of the BBB [14]. In addition, a previous study found that the activation of the MCT1 transporter increases the concentration of lactic acid in the brain, resulting in a decrease in brain pH, which affects normal cellular processes and the physiological function of the brain [4]. Moreover, mitochondrial dysfunction also leads to increased production of lactic acid and decreased brain pH, which subsequently affects the release of neurotransmitters. The increased level of lactate causes an increase in the formation of lactic acid–calcium complexes, which results in a decrease in calcium ion levels and contributes to panic attack symptoms via the upregulation of gamma-aminobutyric acid (GABA) in the dorsomedial hypothalamic nucleus (DMH), which is regulated by the angiotensin-II pathway. Furthermore, the accumulation of lactic acid leads to disturbances in neuronal energy metabolism and brain activity.

### The metabolism and function of lactic acid in the gut

d-Lactic acid is a metabolite of gut microbial fermentation. Numerous gut microorganisms, such as Lactobacillus, Bifidobacterium, Proteus, Eubacteria, anaerobic bacteria, and Enterobacter, participate in the production of d-Lactic acid [15]. Normally, d-lactic acid is rarely absorbed into the circulation because of the lack of enzyme systems in mammals that rapidly degrade d-lactic acid [15]. The occurrence of acute ischemia in the gut causes local bacteria proliferation, which in turn leads to shedding of the intestinal mucosal epithelium and an increase in the paracellular pathways, eventually resulting in an increase in intestinal mucosal permeability and impairment of the biological barrier function of the gut. Furthermore, the synergistic action of gut endotoxins/bacteria and hypoxia can also lead to an increase in the intestinal mucosal permeability via the stimulation of the release of secondary inflammatory mediators, such as various cytokines. The d-lactic acid produced by microorganisms in the gut diffuses predominantly into the blood circulation through the damaged intestinal barrier, which increases the level of d-lactic acid in the blood; in severe cases, this can cause acidosis. Therefore, plasmatic d-lactic acid may be recognized as an effective marker for intestinal ischemia–reperfusion injury. In at least one study, increased plasmatic d-lactic acid was reported in specific pathological conditions, such as short bowel syndrome; moreover, acidosis was also observed [16]. Gut-derived d-lactic acid is eliminated primarily by the liver and kidney during circulation in the blood; however, a small amount of d-lactic acid is transported into the brain through the BBB after binding to the MCT1 transporter [17].

### Lactic acid in the microbiota–gut–brain axis

Lactic acid bacteria (LAB) are a group of bacteria that produce lactic acid from fermentable carbohydrates. LAB are considered to be highly beneficial for health; they activate mucosal function and systematic immunity to fight against infections [18]. Previous studies showed that LAB can affect the composition of gut microbiota, beyond immunomodulatory effects [19, 20]. In the indomethacin-induced gut injury model, lactic acid produced by the probiotic *Lactobacillus casei* causes a decrease in neutrophil infiltration and expression of cytokines, thereby reducing neuroinflammation [21]. However, gut microorganisms can produce a large amount of lactic acid. When the gut barrier is damaged, lactic acid in the gut diffuses into the blood circulation and can cause lactic acidosis. This affects the function of the CNS through the gut–brain axis (nerve, immune, and endocrine pathways) and can cause impairments of neural function, such as ataxia and slurred speech [22]. In addition, it was reported that neurological symptoms in patients with chronic fatigue syndrome may be caused by the excessive absorption of d-lactic acid due to the increased intestinal permeability and expansion of small intestinal bacteria [23]. Intestinal epithelial cells are an important barrier that protects against ectogenic antigens, pathogenic bacteria and their toxins. The health of this barrier is closely related to the incidence and severity of inflammatory bowel disease [24], and lactic acid produced by gut microorganisms plays an important role in the regeneration of intestinal epithelial cells [18]. Taken together, these findings demonstrate that lactic acid is an important intermediate medium between the gut and CNS.

The in-depth understanding of the microbiota–gut–brain axis gained from recent evidence highlights the important role of gut microbiota-derived lactic acid in neuropsychiatric disorders. Previous studies found that *Lactobacillus reuteri*, a LAB that exists in the gut of mammals, upregulated the expression of the neuropeptide hormone oxytocin through the vagus nerve pathway [25]. Increased levels of oxytocin were showed to be associated with the onset of various neuropsychiatric disorders, such as Depression, Anxiety, Autism, and SCZ [26]. Lactobacillus transplantation or lactate administration is effective for improving memory in mice by increasing the level of hippocampal GABA, which is the main inhibitory neurotransmitter in the CNS that participates in the pathogenesis of Anxiety and Depression [27]. Furthermore, another study showed that ingestion of LAB regulated emotional behaviors and central GABA receptor expression in mice via the vagus nerve pathway [25]. Moreover, during conditions of high-intensity stress, the amount of LAB in the stool decreases [28].

## Lactic acid and neuropsychiatric disorders

### Major depressive disorder

MDD is a common mental illness and a primary mood disorder type that is characterized by significant and prolonged depression. MDD is the main cause of disability worldwide. According to the latest report released in 2017, there are approximately 322 million people with MDD worldwide, with a prevalence rate of 4.4%. The prevalence rate of MDD in China is approximately 4.2% [29]. Increasing evidence indicates that lactic acid plays an important role in the pathogenesis of MDD (Table 1). Previously, spectra acquired from the pregenual anterior cingulate cortex using the maximum echo J-resolved spectroscopy protocol in patients with MDD and healthy controls showed a significant increase in the level of lactic acid in MDD patients and was associated with the severity of Depression [30], demonstrating the potential role of lactic acid in the pathogenesis of MDD. Lactic acid produced during exercise is involved in the regulation of brain function and induces an anti-depressant effect [31]. Studies confirm that a single exhaustion task alleviates depressive symptoms in MDD patients, and this improvement may be related to the increased serum concentration of lactic acid [32]. Furthermore, there is a complex relationship between Depression and sleep quality. Other studies revealed that lactic acid in both the blood and brain fluctuates during wake-sleep cycles in mice and increases during rapid eye movement sleep, which suggests that increased lactic acid impacts sleep quality, an essential element in the improvement of depression symptoms; however, the underlying mechanisms are not well understood [33].

**Table 1:** Summary of the mechanism of lactic acid action in disease conditions

Disease	Mechanism
MDD	Accumulation of lactic acid leads to disturbances in neuronal energy metabolism [30]
	Lactic acid activates GPR81 to promote anti-inflammatory effects and inhibit GABAergic neurotransmission [36]
Anxiety	Increased levels of lactic acid cause an increase in the formation of lactic acid-calcium complexes, which leads to a decrease in calcium ion levels [42, 43]
	Lactic acid causes panic attacks via the upregulation of GABA in the DMH region, which is regulated by the angiotensin-II pathway [44]
	Lactic acid activates the GPR81 to promote anti-inflammatory effects [45]
	Lactic acid can selectively promote the expression of genes related to neuroplasticity by enhancing NMDA signals in neurons to facilitate synaptic plasticity and memory formation during a state of anxiety [46]
BD	Apoptosis and reactive oxygen production are caused by mitochondrial dysfunction related to BD [47]
	The accumulation of lactic acid leads to a decreased pH value of the brain, which subsequently affects neuronal activity [49]
SCZ	The accumulation of lactic acid leads to a decreased pH value of the brain, which subsequently affects the release of neurotransmitters [58]
AD	The energy supplied by lactic acid reduces mitochondrial damage mediated by the deposition of Aβ proteins [65, 66]
	The accumulation of lactic acid leads to a decreased pH value of the brain, which subsequently affects brain function [72]
MG	An increase in the level of lactic acid causes an increase in the formation of lactic acid-calcium complexes, which leads to a decrease in calcium ion levels and affects the function of the neuromuscular junction [74]
MS	Mitochondrial dysfunction causes an imbalance in energy metabolism, which drives neuronal degeneration [48, 50]
	Lactic acid influences the level of BDNF proteins [4, 83]

Energy metabolism may be another potential mechanism underlying the involvement of lactic acid in the pathogenesis of Depression. Previous studies showed that an increased level of lactic acid in the cerebrospinal fluid is associated with mitochondrial dysfunction in patients with MDD [34]. Another study in patients with severe Depression also found that mitochondrial dysfunction caused the accumulation of lactic acid, which led to disturbances in neuronal energy metabolism and abnormal brain activity [30]. Lactic acid can directly activate GPR81 to promote anti-inflammatory effects and inhibit GABA-ergic neurotransmission, which affects sleep, learning, and memory. Moreover, it impacts neurotransmission, neurovascular coupling, and neuronal energy metabolism by binding to GPR81 to participate in the regulation of mood disorders [32, 35, 36]. In addition, colonization of the lactic acid-producing bacteria *Bifidobacteria* in mice exerts a significant antidepressant effect by regulating the gut microbiota [37]. Moreover, the biological LAB *Enterococcus faecalis* 2001, was showed to be effective in preventing inflammatory bowel disease-like pathological changes and improving depression-like behaviors in mice by regulating the hippocampal NFκB p65/XIAP pathway [38]. A protective, but not pathogenic, effect of lactic acid on depression was also identified in certain cases, mainly via various epigenetic mechanisms regulated by histone deacetylases [31]. Taken together, lactic acid may be involved in the pathogenesis of Depression through the gut–brain axis.

### Anxiety

Anxiety is a common mental disorder. A considerable number of studies demonstrated a close connection between lactic acid and Anxiety (Table 1). Psychosocial and physical stress can increase anxiety symptoms, accompanied by an increase in blood lactic acid levels [39]. Recent research showed that proliferation of LAB in the gut ferments the sugar content of food and produces high levels of lactic acid, which, if sustained over time, can result in the development of neuropsychiatric disorders [40, 41]. Early in 1967, Pitts and McClure reported that increased levels of serum lactic acid cause an increase in the formation of lactic acid–calcium complexes, which leads to decreased serum calcium ion levels, which are in turn associated with the occurrence of anxiety [42, 43]. Other studies also confirmed that lactic acid intake causes sustained anxious symptoms [39, 43], whereas calcium ion supplements prevent anxiety caused by increased lactic acid [43]. In addition, studies have shown a causative role of lactic acid in panic attacks via the upregulation of GABA in the dorsomedial hypothalamic nucleus region, which is regulated by the angiotensin-II pathway [44]. However, lactic acid can inhibit adenylate cyclase 5 by activating GPR81, which results in a decrease in cyclic adenosine 3′,5′-monophosphate expression, and this leads to a decrease in PKA expression and a reduction in the inflammatory factor, which contributes to the relief of anxiety-like behaviors [45]. In addition, lactic acid selectively promotes the expression of genes related to neuroplasticity (e.g., activity-regulated cytoskeleton-associated protein and BDNF) by enhancing NMDA receptor signals in neurons to promote synaptic plasticity and memory formation in patients with Anxiety [46].

### Bipolar disorder

Bipolar disorder (BD) is a major mental illness that is characterized by alternating episodes of Mania and Depression [47, 48]. Previous studies found significantly elevated lactic acid levels in the brain of patients with BD without concurrent alteration of peripheral lactic acid level, and that blood lactic acid levels increase after treatment for BD [49], which suggests a regulatory role of lactic acid in the pathogenesis of BD (Table 1). However, the underlying mechanisms of this role are unclear. Mitochondrial dysfunction plays a key role in the pathophysiology of BD via apoptosis and reactive oxygen production [50], and the expression of lactic acid is a classical biological indicator for evaluating mitochondrial dysfunction [48]. Kato proposed for the first time that the pathogenesis of BD is related to mitochondrial dysfunction by showing that pathological conditions in BD increase anaerobic energy metabolism and the level of lactic acid in the body [47], followed by an accumulation of lactic acid and a decrease in brain pH, which subsequently affects neuronal activity [47, 50].

### Schizophrenia

SCZ is a severe psychosis that usually has a subacute or chronic onset during youth and middle age. It is characterized by hallucinations, thinking disorders, impaired emotion and motivation, and cognitive dysfunction [51]. Previous studies found that higher levels of LAB in the gut of patients with severe SCZ are positively correlated with symptom severity [52, 53]. Other studies also found that *Lactobacillus gasseri* is more abundant in the oral cavity of patients with SCZ than of healthy controls [54, 55]. It is well established that LAB as probiotics promote health and suppress inflammation [56]. Therefore, it is surprising that an increased level of LAB is associated with greater severity of symptoms in patients with SCZ. In addition to the above, effects of lactic acid on SCZ, recent studies indicated that lactic acid-related energy metabolism in the brain is related to the pathophysiology of SCZ, and increased levels of lactic acid were identified in the brain of SCZ patients [57]. These increased lactic acid levels are associated with SCZ-related energy metabolism dysfunction [58] (Table 1). Increased levels of lactic acid in the brain are primarily caused by disturbances in the transformation of the TCA cycle and oxidative phosphorylation, as well as glycolytic energy metabolism, due to extensive mitochondrial dysfunction and increased oxidative stress damage. Mitochondrial dysfunction in SCZ leads to an increase in the production of lactic acid and a decrease in brain pH, which subsequently affects neurotransmitter release [58, 59]. Therefore, improving mitochondrial energy metabolism is effective in alleviating cognitive and neural functions in patients with SCZ [51].

Synaptic dysfunction was also reported in patients with SCZ [57], and studies suggest the regulation of excitatory synapses as a potential pathogenic mechanism of SCZ [60]. Synapse maintenance and neuronal energy metabolism are essential for synaptic neurotransmission [57]. Moreover, various studies showed that lactic acid is necessary for the maintenance of synaptic function [61], and the energy metabolism that maintains normal synaptic function is abnormal in SCZ [57]. These findings indicate that lactic acid is involved in the maintenance of normal synaptic and neuronal function in SCZ [57, 61].

### Alzheimer’s disease

AD is a neurodegenerative disease characterized by progressive cognitive impairment and dementia. The deposition of amyloid β-protein (Aβ) plaque is the main pathological feature of AD, and results in damage to neurons and axons/synapses [62]. Lactic acid is essential for memory formation [8]. Previous studies reported reduced secretion of lactic acid in the astrocytes of AD patients [63], which may contribute to the pathophysiology of AD. Lactic acid produced in astrocytes is an important substrate for neuronal energy metabolism [64]. In AD patients, glucose metabolism is inhibited because of decreased uptake of neuronal glucose, decreased activity of the electron transport chain, and mitochondrial dysfunction [62]. In this case, the lactic acid produced during glycolysis ensures sufficient energy supply in the brain and reduces mitochondrial damage mediated by the deposition of Aβ protein [65, 66] (Table 1). Studies demonstrated that lactic acid is an effective neuroprotective agent, and the administration of lactic acid helps maintain neuronal activity during glucose deprivation [67]. The transportation of lactic acid from astrocytes to neurons is key to the formation of long-term memory [8]. Moreover, the accumulation of lactic acid in the brain promotes the deposition of Aβ protein [68–71], and the excessive transmission of lactic acid into neurons leads to a decrease in the pH value, resulting in a failure of mitochondrial function and apoptosis, which ultimately impacts brain function [72]. The role of lactic acid in the pathogenesis of AD appears to be bidirectional. The concept of AD is the progression from “brain disease” to “metabolic-cognitive syndrome” [73], and the role of lactic acid in this process warrants further exploration.

### Myasthenia gravis

MG is an autoimmune disease caused by transmission dysfunction at the neuromuscular junction and is characterized by partial or systemic skeletal muscle weakness and being prone to fatigue. These symptoms are aggravated by activity and relieved following rest. In the early twentieth century, Walker et al. reported the presence of lactic acid in the blood of MG patients and suggested that it contributed to the development of myasthenia [74] (Table 1). Subsequent studies focusing on the underlying mechanisms revealed that this pathological change is due to the combination of lactic acid and calcium [75], which reduces ionized calcium and total serum calcium at the neuromuscular junction [74]. This reduction in serum calcium decreases the release of acetylcholine [74, 76] and affects the function of the neuromuscular junction. These processes may be underlying mechanisms of myasthenia induced by lactic acid in patients with MG [74]. In another study, increasing serum calcium promoted the release of acetylcholine and relieved related myasthenia symptoms caused by lactic acid intake in patients with MG [74]. In addition, massive amounts of lactic acid produced in patients with MG result in the exacerbation of symptoms [77], which demonstrates the adverse effects of lactic acid on patients with MG.

### Multiple sclerosis

MS is an inflammatory disease that damages myelinated axons of the CNS [78]. In recent years, there is increasing evidence that suggests that the pathogenesis of MS is closely related to mitochondrial dysfunction and oxidative damage. Mitochondrial dysfunction causes an imbalance of energy metabolism, which drives neuronal degeneration and promotes the development of MS [79]. A previous study found a significant increase in the level of lactic acid in the cerebrospinal fluid of MS patients, and treatments that improve mitochondrial function help postpone the progression of MS symptoms [79], which demonstrates that mitochondrial dysfunction and the related lactic acid-driven energy metabolism are associated with the physiopathological mechanisms underlying MG [48, 50] (Table 1). Compared with healthy controls, patients with MS have higher resting blood lactic acid levels, which can be restored with moderate-intensity exercise therapy but not with high-intensity exercise therapy [80]. This restorative effect of exercise may result from the fact that lactic acid is typically removed by muscle gluconeogenesis and oxidation or transported to the blood and filtered and removed by the kidneys [81, 82]. However, under high-intensity exercise, because of hypoxia and the related increase in glycolysis, lactic acid increases rapidly and cannot be metabolized promptly, which eventually causes the accumulation of lactic acid. Numerous studies also found that lactic acid plays an important role in the muscle–brain endocrine circuit, in which the skeletal muscles secrete myokines or express muscle factors to affect brain function directly or indirectly by influencing the level of BDNF proteins [4, 83]. In addition, the energy metabolism in the CNS is correlated with the severity of MS symptoms [79].

Regarding the microbiota–gut–brain axis, probiotic treatments, which include two lactobacilli and two bifidobacteria, were showed to effectively modulate disease symptoms in both experimental autoimmune MG and experimental autoimmune encephalomyelitis models [84–86]. Moreover, administration of lactobacillus to patients with MS improves expanded disability status scale scores and symptoms of Depression and Anxiety [78, 87].

## Summary and future perspectives

In-depth investigations into lactic acid revealed that the role of lactic acid in the body is diverse. As pairs of enantiomers in the human body, DL lactic acid combines with GPR81 to regulate neural and network activity. However, l-lactic acid primarily acts as an energy metabolism substrate to provide energy for neuronal activity and support protein synthesis during learning and memory. d-lactic acid blocks the uptake of l-lactic acid by neurons and impairs memory. Lactic acid is not only a metabolite produced during exercise that causes fatigue symptoms but also an important contributor to energy metabolism that affects systemic physiological functions. Furthermore, it participates in the regulation of brain function as a signal molecule, which affects the development of neuropsychiatric diseases. In the brain, astrocytes produce lactic acid to supply energy for neurons by transporting lactic acid from astrocytes to neurons and maintaining normal neuronal function. However, the disruption of lactic acid metabolism leads to the accumulation of lactic acid and an insufficient energy supply in the brain, which results in brain dysfunction and the onset of neuropsychiatric diseases. Insufficient lactic acid production results in an inadequate neuronal energy supply, which affects normal physiological responses and results in brain dysfunction. Conversely, the buildup of lactic acid can lead to abnormal activity in brain areas that cause lactic acid to rise, which leads to brain dysfunction. Therefore, an in-depth understanding of the molecular mechanisms by which lactic acid regulates brain function is of great value for the early diagnosis and prevention of neuropsychiatric diseases. Although associations between lactic acid and neuropsychiatric diseases were reported in previous research, the underlying pathogenic mechanisms remain unclear. To date, studies confirmed that lactic acid affects the blood–brain and intestinal barriers. Moreover, as a signal molecule, lactic acid was showed to regulate brain behavior and disease via the microbiota–gut–brain axis and serve as a key target for understanding how the gut microbiota regulates brain behavior via the gut–brain axis. The findings in this review show great promise for the analysis of the effects of lactic acid on brain function and the regulation of neuropsychiatric diseases, and provide new avenues for the prevention and treatment of neuropsychiatric diseases.

## Data Availability

Not applicable.
